# Complete Genome Sequences of Human Immunodeficiency Type 1 Viruses Genetically Engineered To Be Tropic for Rhesus Macaques

**DOI:** 10.1128/genomeA.01063-17

**Published:** 2017-09-28

**Authors:** Masako Nomaguchi, Naoya Doi, Takaaki Koma, Akio Adachi

**Affiliations:** a Department of Microbiology, Tokushima University Graduate School of Medical Science, Tokushima, Japan; b Department of Microbiology, Kansai Medical University, Osaka, Japan

## Abstract

We have constructed two human immunodeficiency type 1 (HIV-1) derivatives, CXCR4 tropic and CCR5 tropic, that replicate in rhesus macaques. They are genetically engineered to be resistant to macaque restriction factors against HIV-1, including TRIM5α, APOBEC3, and tetherin proteins. The two HIV-1 variants described here are fundamental clones aiming for rhesus infection studies of HIV-1.

## GENOME ANNOUNCEMENT

Human immunodeficiency type 1 (HIV-1) is not infectious at all for a variety of small animal species widely used for experimental virus infections. Moreover, significant portions of basic and applied studies on HIV-1 have been hampered due to the lack of appropriate primates susceptible to this virus (1–3). Thus, *in vivo* model studies using HIV-1 itself and its susceptible hosts are currently thought to be implausible or extremely difficult. However, regardless of the above striking property of HIV-1, researchers, including us, have made efforts to establish primate models for HIV-1 infection by exquisitely modifying the HIV-1 genome, not by changing or genetically manipulating host animals. A common theme is the generation of macaque-tropic HIV-1s that are able to infect various species of macaques (4–9).

HIV-1 is tropic for chimpanzees and humans and causes AIDS almost only in humans. Macaques, frequently used for primate studies of experimental virus infections, are not susceptible to HIV-1 at all. Although not completely elucidated yet, the remarkably narrow species tropism of HIV-1 has resulted mainly from cellular restriction factors against the virus (10–12), especially from TRIM5α and APOBEC3 proteins. Viral proteins that interact with and inactivate the TRIM5α and APOBEC3 proteins have been demonstrated to be Gag capsid and Vif, respectively. We and others have pioneered the genetic construction of HIV-1 with macaque tropism and successfully generated HIV-1 derivatives tropic for macaque cells by replacing the *gag* capsid and *vif* sequences with those of SIVmac239, a standard clone that replicates well in rhesus macaques and induces AIDS in infected animals (4, 5). These prototype macaque-tropic HIV-1 clones encode a complete Vif protein derived from SIVmac239 that is capable of degrading macaque APOBEC3 proteins, thus fully inactivating their antiviral activities (4, 5). However, macaque-tropic HIV-1 clones at that stage replicated much more poorly than the SIVmac239 clone, probably due to the insufficient modifications of the Gag capsid region. As surrogate animal models for studies on HIV-1 replication, HIV-1 pathogenesis, drug evaluation, and/or vaccine development, the rhesus macaque/SIVmac system has been frequently and successfully used. Taken together, we and others have recently generated novel versions of rhesus macaque-tropic HIV-1 (HIV-1rmt) clones through further alterations of the *gag* capsid region by various genetic methods (13, 14) and have obtained viruses that replicate in rhesus cells at a level comparable to that of SIVmac239 (13–15). We have also modified the viral *vpu* gene (14) to inactivate another cellular restriction factor tetherin that can contribute to the species tropism of HIV-1 (10–12). Indeed, our two new HIV-1rmt clones thus generated (14, 16) replicated considerably in rhesus macaques (our unpublished data).

We sequenced full genomes of the two plasmid DNA clones, designated pMN4/LSDQgtu and pMN5/LSDQgtu, using an ABI Genetic Analyzer 3130xl (Thermo Fisher Scientific, USA) with a series of primers. Viruses derived from pMN4/LSDQgtu and pMN5/LSDQgtu are CXCR4 and CCR5 tropic, respectively, and are thus useful for various experimental analyses. These two macaque-tropic proviral clones lay valuable foundations for future HIV-1 model studies in primates.

### Accession number(s).

The complete genome sequences of HIV-1rmt clones pMN4/LSDQgtu and pMN5/LSDQgtu have been deposited in DDBJ/EMBL/GenBank under accession numbers LC315178 and LC315179, respectively.
